# Protective effects of intermittent hypoxia on brain and memory in a mouse model of apnea of prematurity

**DOI:** 10.3389/fphys.2015.00313

**Published:** 2015-11-04

**Authors:** Myriam Bouslama, Homa Adle-Biassette, Nelina Ramanantsoa, Thomas Bourgeois, Bieke Bollen, Olivier Brissaud, Boris Matrot, Pierre Gressens, Jorge Gallego

**Affiliations:** ^1^Inserm, U1141, Robert Debré HospitalParis, France; ^2^Paris Diderot-Paris 7 UniversityParis, France; ^3^Department of Pathology, Lariboisière HospitalAP-HP, Paris, France; ^4^Laboratory of Biological Psychology, University of LeuvenLeuven, Belgium; ^5^Neonatal Intensive Care Unit, Bordeaux University HospitalBordeaux, France

**Keywords:** sleep disordered breathing, neurogenesis, brain injury, cognitive dysfunction, control of breathing

## Abstract

Apnea of prematurity (AOP) is considered a risk factor for neurodevelopmental disorders in children based on epidemiological studies. This idea is supported by studies in newborn rodents in which exposure to intermittent hypoxia (IH) as a model of AOP significantly impairs development. However, the severe IH used in these studies may not fully reflect the broad spectrum of AOP severity. Considering that hypoxia appears neuroprotective under various conditions, we hypothesized that moderate IH would protect the neonatal mouse brain against behavioral stressors and brain damage. On P6, each pup in each litter was randomly assigned to one of three groups: a group exposed to IH while separated from the mother (IH group), a control group exposed to normoxia while separated from the mother (AIR group), and a group of untreated unmanipulated pups left continuously with their mother until weaning (UNT group). Exposure to moderate IH (8% O_2_) consisted of 20 hypoxic events/hour, 6 h per day from postnatal day 6 (P6) to P10. The stress generated by maternal separation in newborn rodents is known to impair brain development, and we expected this effect to be smaller in the IH group compared to the AIR group. In a separate experiment, we combined maternal separation with excitotoxic brain lesions mimicking those seen in preterm infants. We analyzed memory, angiogenesis, neurogenesis and brain lesion size. In non-lesioned mice, IH stimulated hippocampal angiogenesis and neurogenesis and improved short-term memory indices. In brain-lesioned mice, IH decreased lesion size and prevented memory impairments. Contrary to common perception, IH mimicking moderate apnea may offer neuroprotection, at least in part, against brain lesions and cognitive dysfunctions related to prematurity. AOP may therefore have beneficial effects in some preterm infants. These results support the need for stratification based on AOP severity in clinical trials of treatments for AOP, to determine whether in patients with moderate AOP, these treatments are beneficial or deleterious.

## Introduction

Apnea of prematurity (AOP) indicates a cessation of breathing lasting longer than 15–20 s and/or accompanied by oxygen desaturation and bradycardia in premature newborns (Zhao et al., 2011). The incidence of AOP varies with the degree of prematurity, from 7% at 34–35 weeks of gestational age to 15% at 32–33 weeks, 54% at 30–31 weeks, and nearly 100% at < 29 weeks and in babies weighing less than 1000 g at birth (Zhao et al., 2011). AOP generally resolves at around term-equivalent age (Carroll and Agarwal, 2010). However, sleep-breathing disorders are more prevalent in children born preterm (Raynes-Greenow et al., 2012). AOP severity is correlated with the degree of neurodevelopmental disorders (Martin and Wilson, 2012), although the specific contribution of AOP to these disorders is unknown due to the confounding effect of numerous prematurity-related comorbidities such as brain injury, bronchopulmonary dysplasia (Gozal et al., 2012; Gozal, 2013), and the potential psychological impact of physical separation during the first weeks of life (Welch et al., 2014). Whether the best approach to AOP is watchful waiting, or whether it must be actively treated to prevent adverse neurodevelopmental effects remains a major unresolved issue.

A popular model in which to assess the developmental effects of AOP consists of exposing newborn rodents to repeated short bouts of hypoxia (intermittent hypoxia, IH). This method mimics hypoxia-reoxygenation sequences, considered the main cause of AOP-related morbidity. In previous studies, neonatal IH has been shown to increase apoptosis in the cortex and hippocampus (Gozal et al., 2001a) and impair hippocampal neuronal function (Douglas et al., 2007), dopaminergic signaling (Decker et al., 2005; Kheirandish et al., 2005) and myelination in the corpus callosum and white matter (Cai et al., 2012; Juliano et al., 2015). IH also appears to cause sensorimotor deficits, locomotor hyperactivity and memory impairments (Row et al., 2002; Decker et al., 2003, 2005; Kheirandish et al., 2005; Juliano et al., 2015). However, the marked hypoxia used in these studies, especially the duration and frequency of IH exposure (for example, 2–3 weeks, 60 events/h) may not fully reflect the broad spectrum of AOP severity. Uncertainties about the effects of less severe IH on development are further amplified by studies of conditions other than apnea, in which neonatal hypoxia has proven to be neuroprotective through a process known as preconditioning (Almendros et al., 2014). These results invite an assessment of whether hypoxia can exert neuroprotective effects under conditions that mimic moderate AOP, a question that has not been addressed to date.

In this study, we hypothesized that moderate IH would protect the neonatal mouse brain against the detrimental effects of behavioral stressors and brain injury. The stressor used was separation from the mother, an event known to impair brain development in rodents (Fabricius et al., 2008; Aisa et al., 2009). In a separate experiment, we combined maternal separation with excitotoxic brain insults mimicking those seen in preterm infants (Bouslama et al., 2007). In both experiments, pups were exposed to moderate IH or air from postnatal day 6 (P6) to P10. Because P6 in rodents corresponds to about 32 weeks of gestation in humans and P7–P10 to term (Semple et al., 2013), this IH exposure period in mice replicated the period during which AOP occurs in humans. We then evaluated development using behavioral, and histological criteria.

## Materials and methods

### Animals

Experiments were performed on newborn (*n* = 373) male and female Swiss mice (Janvier, Le Genest Saint Isle Saint Berthevin, France). The mice were housed under controlled temperature and humidity conditions (22–25°C and 55% humidity) and allowed free access to food and water. A 12-h light-dark cycle was maintained with lights on between 8 a.m. and 8 p.m.

All experimental protocols were approved by local (Bichat-Robert-Debré) and national committees (Ministère de l'Enseignement Supérieur et de la Recherche—Direction Générale pour la Recherche et l'Innovation), in accordance with the European Communities Council Directive of September 22, 2010 (2010/63/EU for animal care). Every effort was made to minimize animal suffering. The pups were killed by decapitation and the adult mice were killed by cervical dislocation upon completion of the behavioral tests.

### Intermittent hypoxia

**Figure 1:**
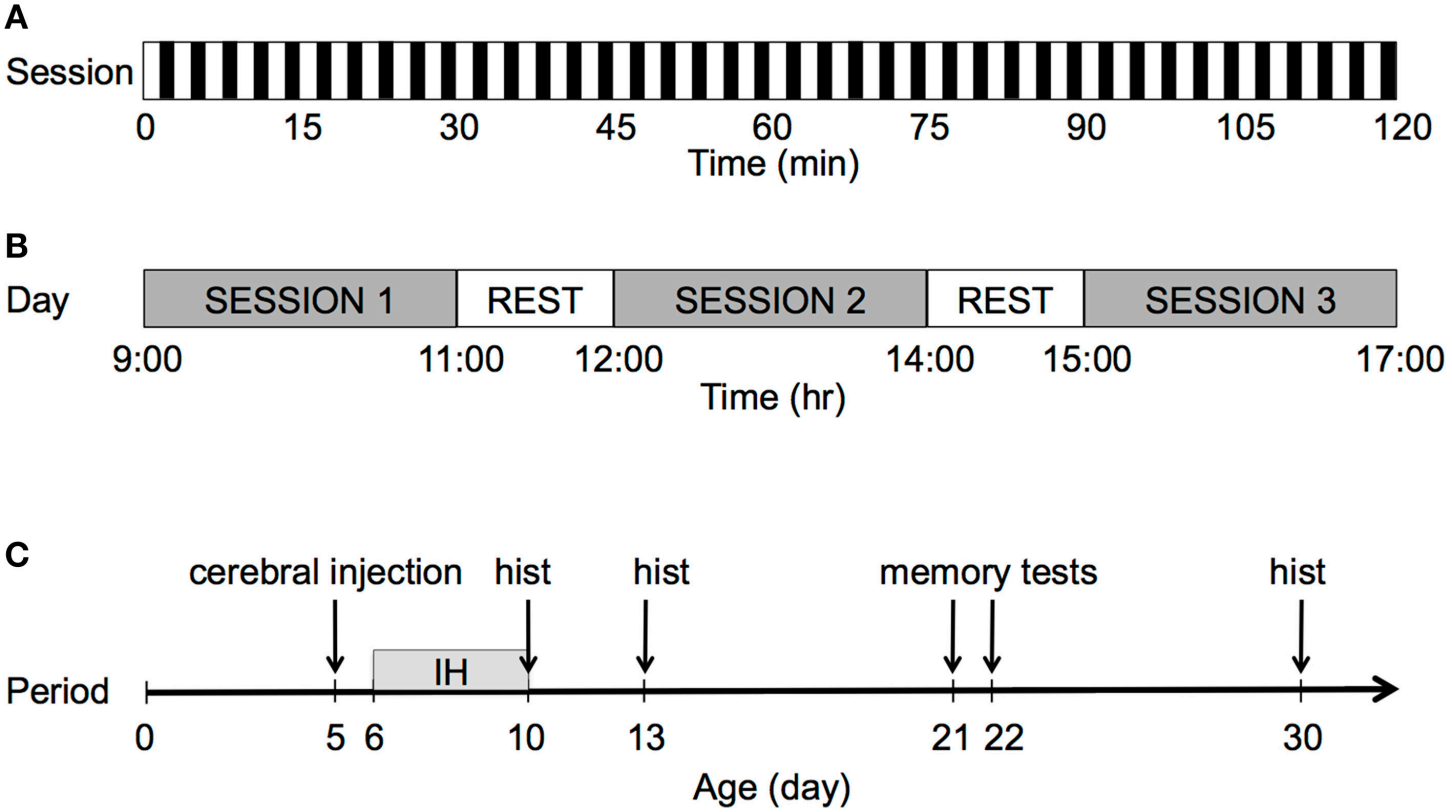
**Graphical representation of the IH protocol. (A)** Each session lasted 2 h with a frequency of 20 events/h. For each hypoxic event, after 90 s of normoxia, pure N_2_ was injected into the chamber for 30 s, which decreased F_I_O_2_ from 20.9 to 8%. F_I_O_2_ was maintained at 8% for 60 s. **(B)** The pups were exposed to IH during three 2-h sessions per day. During rest periods, the pups were returned to their dam for 1 h. After completion of the third session, all pups were returned to their dam until the following day. **(C)** The pups were exposed to IH over 5 consecutive days, from P6 to P10; hist: histology.

Figure 1 describes the IH protocol. On P6, each pup in each litter was randomly assigned to one of three groups: a group exposed to IH and maternal separation (IH group), a group exposed to normoxia and maternal separation (AIR group), and a group of untreated unmanipulated pups left continuously with their mother until weaning under standard housing conditions (UNT group). The UNT group controlled for the effects of maternal separation on development.

The pups were exposed to IH without their dam in a 100-ml plastic chamber equipped with heating wires to maintain body temperature within the physiological range (30°C) and with a fan to ensure constant wash-out kinetics and a reproducible hypoxic profile. The flow through the chamber was controlled by programmable valves (Bürkert, Triembach au Val, France) and flow regulators (Vögtlin, Aesch, Switzerland), which controlled the oxygen concentration in the chamber by switching between room air at 8 L/min and nitrogen (N_2_) at 2.5 L/min). After 90 s of normoxia, pure N_2_ was injected into the chamber for 30 s, which reduced the F_I_O_2_ from 20.8 to 8%. F_I_O_2_ was maintained at 8% for 60 s by interrupting the gas flow through the chamber. Gas concentrations were monitored continuously using an O_2_/CO_2_ analyzer (Arelco, Fontenay sous Bois, France).

Pups were exposed to IH or air during three 2-h sessions per day, over five consecutive days from P6 to P10. After the first and second daily sessions, the pups were returned to their dam for 1 h. After completion of the third session, the pups were returned to their dam and left undisturbed until the following day.

### Arterial oxygen saturation and heart rate

To characterize the IH protocol physiologically, we measured arterial oxygen saturation (SpO_2_) and heart rate (HR) in separate groups of pups exposed to the IH (*n* = 9) or AIR (*n* = 5) protocol, from P6 to P10, using the MouseOx Oximeter (STARR Life Sciences, Oakmont, PA, USA) as described previously (Cai et al., 2011). The pups were gently restrained inside the hypoxic or normoxic chamber. The collar clip (XS size) with a sensor probe (4 mm) was placed on the temporal regions of the head. Only error code-free data analyzed by the MouseOx software were included in our analysis.

### Memory tests

Two tests, the object recognition task (ORT) and the object location task (OLT) were used to assess memory. These tests are based on the natural tendency to explore either unfamiliar objects (ORT) or familiar objects placed at unfamiliar locations (OLT). Both tasks involve working memory and attention (Li et al., 2011).

The ORT then the OLT were conducted on P21 and P22 as described previously (Li et al., 2011) with minor adaptations. Twenty-four hours before the task, the mice were familiarized with the testing chamber (36 x 36 x 10 cm) located in a brightly lit testing room, for 1 h. Both tasks consisted of a 3-min training session followed by a 3-min test session, 30 min apart. In the training session of both the ORT and the OLT, the mouse was placed in the center of the chamber, which contained two identical objects (ORT: red plastic bottle caps, diameter 34 mm, height 1.7 cm; OLT: plastic parallelepipeds 1.2 × 10.5 × 2 cm) securely fixed to the floor at two adjacent corners of the chamber. The mouse was returned to its home cage after the training session for 30 min. Then, it was replaced in the center of the chamber for the test session. In the ORT test phase, one of the two objects was replaced by its identical copy (to avoid olfactory trails) and the second object by another object of different shape and color (yellow rubber bulb, diameter 25 mm, height 5 cm). In the OLT test phase, the two objects were replaced by their identical copies, one of which was placed in the same position as during the training session and the other moved to the adjacent corner. All object combinations and locations were used in a balanced manner to reduce potential biases due to preference for specific locations or objects. For both tests, the experimenter measured the exploration time defined as the time spent directing the nose to the object at a distance of less than 2 cm or touching the object with the nose. Turning around, climbing over, or sitting on the object were not recorded as exploration (Bevins and Besheer, 2006). The preference index (in percentage) was defined as the time spent exploring the new (or displaced) object divided by the total time spent exploring the two objects. The discrimination index was defined as the absolute difference between the time spent exploring the new (or displaced) object and the time spent exploring the other object. The objects and box were cleaned after each trial with 70% ethanol to prevent a build-up of olfactory cues. The tests were conducted by assessors who were blinded to treatment group.

### Brain lesions

Excitotoxic brain lesions were induced by injecting 10 μg of ibotenate (Tocris Bioscience, Bristol, UK) into the brain on P5 as described previously (Bouslama et al., 2007). Ibotenate acts selectively on *N*-methyl-*D*-aspartic acid (NMDA) and metabotropic receptors. Briefly, the pups were anesthetized with isoflurane, kept under a warming lamp, and given an ibotenate injection into the neopallial parenchyma, using a 25-gauge needle on a 50 μL Hamilton syringe mounted on a calibrated microdispenser. The needle was inserted 2 mm under the external surface of scalp in the frontoparietal area of the right hemisphere, 1 mm from the midline in the lateral–medial plane and 1.5 mm anterior to the bregma in the rostrocaudal plane. Two 1-μL boluses were injected at 20-s intervals.

The pups were killed on P10. The brains were immediately fixed in 4% formalin. After embedding in paraffin, we cut 16 μm-thick coronal sections and stained every third section with cresyl violet. Previous studies showed close correlations between maximal excitotoxic lesion sizes along the lateral-medial and fronto-occipital axes (Gressens et al., 1997; Husson et al., 2002). We therefore cut serial sections of the entire brain in the coronal plane. This permitted an accurate and reproducible determination of the sagittal fronto-occipital diameter, which was equal to the number of sections in which the lesion was present multiplied by the thickness of the coronal sections. We have previously reported that this estimate is strongly correlated with the extent of the lesion along the mediolateral axis and the radial axis in the coronal plane, thus providing an accurate and reproducible estimate of lesion volume (Gressens et al., 1997; Husson et al., 2002). We used this linear measure as an index of lesion volume.

### Immunohistochemistry

Immunohistochemistry was performed to assess cell proliferation on P13, P21, and P30 using: bromodeoxyuridine (BrdU, BD Biosciences); NeuroD (Santa Cruz, Sc-1084, 1:100 on P21 only), a marker for newly generated dentate gyrus granules; doublecortin (DCX, Abcam, ab18723, 1:200), a marker for young postmitotic neurons, to assess neural proliferation and differentiation in the hippocampal dentate gyrus; and activated caspase 3 (rabbit, Cell Signaling), to assess cell death. For BrdU immunohistochemistry, animals received a single intraperitoneal BrdU injection (50 mg/Kg dissolved in saline) on P10, just after IH exposure. For all markers, the mice were killed by cervical dislocation and the brains were excised, fixed in formalin, and embedded in paraffin. The paraffin-embedded tissue blocks were cut in 20-μm sections, which were deparaffinised with xylene and rehydrated with ethanol. Peroxidase activity was blocked with 10% H_2_O_2_ and the sections were treated with citrate buffer (pH 6.0 at 96°C for 30 min), cooled for 20 min, and incubated in blocking buffer (composed of 20% of serum depending on the species in whom the secondary antibodies were raised, and 0.2% Triton X-100 in PBS) for 1 h at room temperature. Sections were incubated overnight with mouse antibody against BrdU, rabbit polyclonal antibody against DCX or NeuroD then 2 h with donkey anti-rabbit or anti-goat antibody (Invitrogen, Alexa fluor® 1:400). The nuclei were visualized with 49-6-diaminodino-2-phenylindole (DAPI). NeuroD and BrdU immunostainings in parallel sections were performed and visualized using Vectastain Universal Elite ABC Kit. For BrdU labeling, after treatment with H_2_O_2_ and citrate buffer, sections were incubated in 2 N HCl at 37°C for 30 min, rinsed in PBS, and incubated in blocking buffer. The sections were incubated overnight with mouse anti-BrdU (Boehringer-Mannheim, 1:100), and immunolabelings were visualized using the Vectastain Universal Elite ABC Kit. Neuronal density was calculated as their counts per unit area (μm^2^). Caspase-3 immunohistochemistry were carried out for apoptosis assessment in the Ventana BenchMark platform at 1/200 dilution.

Finally, immunocytochemistry for glucose transporter 1 (GLUT-1) was used to assess vascular density in the somatosensory cortex overlying the hippocampus on P30. Cerebral vessels were identified by GLUT-1 immunostaining and vessel density was quantified by counting the number of GLUT-1 positive walls per field at 200X magnification in four cross-sections using a 4000 × 4000 pixel image as described (Olivier et al., 2007), with the ImageJ software package (http://rsbweb.nih.gov/ij/). A potential limitation of this method is that hypoxia is known to increase GLUT-1 expression, leading to greater staining intensity in hypoxia-exposed (Kanaan et al., 2006). However, the vessels are clearly observable despite potential variations in GLUT1 intensity, as previously discussed (Kanaan et al., 2006).

### Cell density

For BrdU and NeuroD cell density, four sections were sampled at 80-μm intervals, starting at -2.40 to Bregma. For each section, the outline of the granule cell layer was traced at 20x and the cells were counted in the entire area of the dentate gyrus, using StereoInvestigator software (MicroBright-Field Europe, Magdeburg, Germany) and a Leica microscope modified by attachment to the stage of a Heidehain VRZ403 electronic length gauge measuring vertical movement through the section. The height of the counting frame (20 × 20 μm) or dissector was 9 μm. Sections had a mean thickness of 14 μm and a guard zone of 3 μm at the top and bottom. Before cell counting, all sections were examined to ensure completeness of immunolabeling throughout the thickness. Sections had a mean thickness of 14 μm. Cells were counted in juxtaposed counting frames at a magnification of 100. *DCX-labeled neurons were too dense to be individually identified by conventional immunoenzymatic labelings*. To assess the density of DCX-positive cells in the dentate gyrus, mosaic scans of the entire surface of the dentate gyrus were obtained using a 40x lens EC Plan-Neofluar oil-immersion lens (numerical aperture 1.3) of a Zeiss Axio Observer inverted microscope (Carl Zeiss, Jena, Germany) equipped with an AxioCam MRm camera. Cell density was established using ImageJ software. For activated caspase-3 immunohistochemistry, the density of labeled nuclei was established in 3 sections. For each section, the outline of the granule cell layer was traced at 20x using ImageJ software (imagej.nih.gov/ij/). The number of the labeled nuclei was counted in the entire area of the dentate gyrus, at x400 magnification. All analyses were conducted by assessors who were blinded to treatment group.

### Statistical analysis

Parametric analyses were performed using one or Two-Way ANOVAs with treatment group as the main factor in all analyses and phase (training vs. test, as a repeated factor) in the analyses of the ORT and OLT. *Post-hoc* paired comparisons were performed using Fisher's least significant difference (LSD) tests (shown in the figures) when main effects or interactions were significant. Litter and sex had no significant effects, either as a main effect or in interaction with treatment, and will not be mentioned further. Values are means (±standard deviations, SD) in the text and means (±standard errors of the means, SEM) in the figures. Statistical tests were conducted using StatView 5 (Abacus Concepts, Berkeley, CA) and Prism (GraphPad Software, La Jolla, CA).

## Results

### SpO_2_ and heart rate responses to intermittent hypoxia

**Figure 2:**
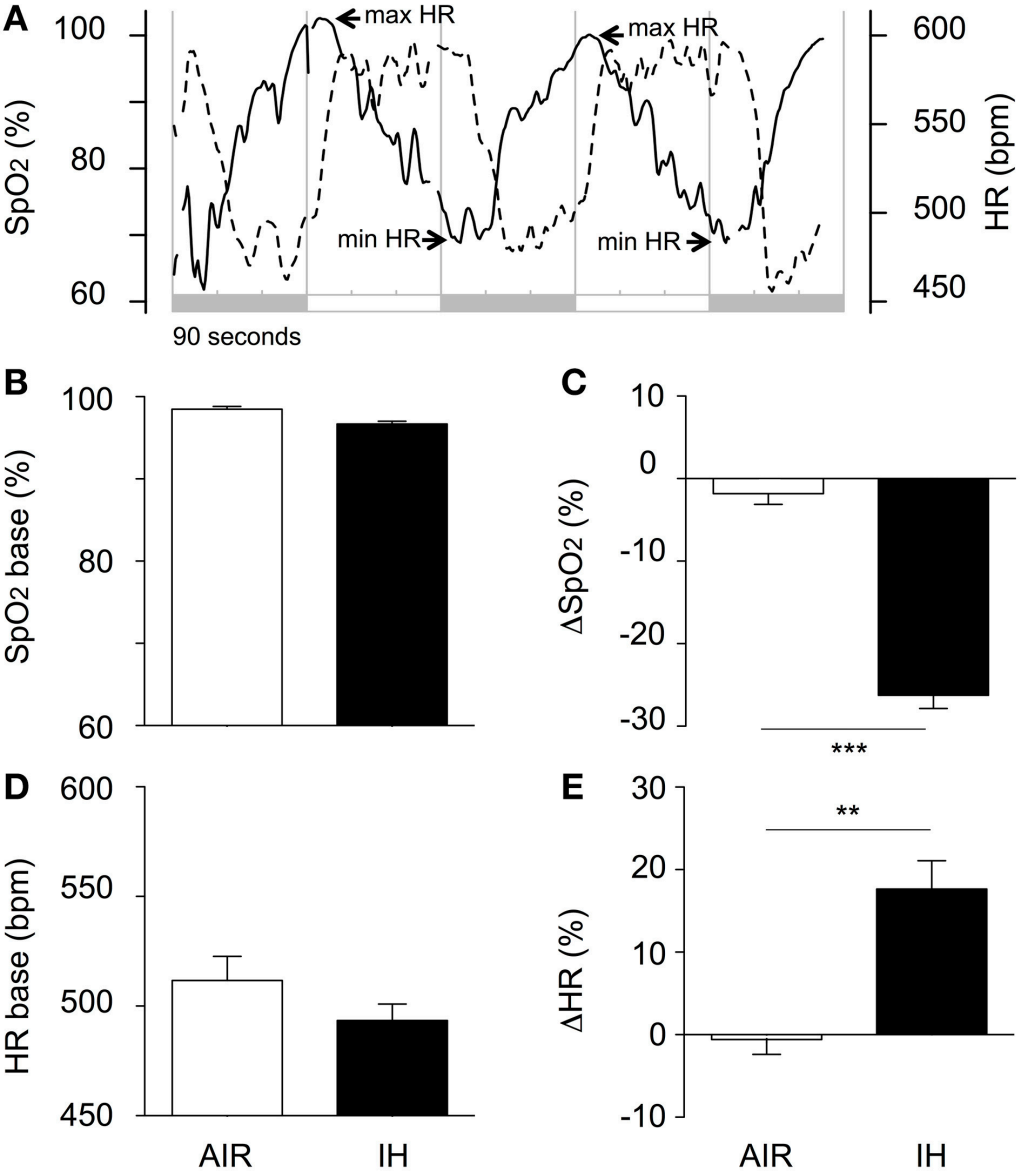
**Acute changes in oxyhaemoglobin saturation (SpO_2_), and heart rate (HR) during IH or air exposure**. **(A)** Illustrative individual traces of SpO_2_ (dotted line) and HR (solid line) in a pup from the IH group. The shaded areas indicate 90-s hypoxic stimuli alternating with 90-s air exposures. **(B)** Baseline SpO_2_ values; **(C)** Hypoxia was associated with a marked decrease in SpO_2_; **(D)** Minimal HR values were used as estimates of baseline values. The difference between the IH and AIR groups was not significant (*p* = 0.181); **(E)** Hypoxia was associated with a moderate increase in HR. The minimal and maximal SpO_2_ values were calculated as the mean values over the last 30 s of hypoxia and air, respectively. The minimal and maximal HR values were calculated as the mean value over the 30-s period following each switch from air to hypoxia and from hypoxia to air, respectively. The percentage responses to hypoxia of SpO_2_ (ΔSpO_2_, **C**) and HR (DHR, **E**) were calculated as 100·(min SpO_2_-max SpO_2_)/max SpO_2_ and 100·(max HR-min HR)/min HR, respectively. SpO_2_ and HR data were pooled over days and sessions, from P6 to P10, as these factors had no significant effect on either variable. Values are group means ± SEM. ^***^p < 0.001, ^**^p < 0.01.

In response to a decrease in F_I_O_2_, SpO_2_ decreased and HR increased (Figure 2A). The hypoxia-induced HR increase was delayed by about 30 s after the onset of hypoxia (Figure 2A, min arrows) and lasted less than 30 s after the initiation of re-oxygenation (Figure 2D, max arrows), after which HR decreased to the normoxic value. Hypoxia decreased SpO_2_ from 96.7% ± 1.0 to 70.4% ± 4.7, i.e., by 27% (Figures 2B,C), and increased HR from 493 ± 22 bpm to 577 ± 39 bpm, i.e., by 17% (Figures 2D,E).

### Intermittent hypoxia improved memory indices

**Figure 3:**
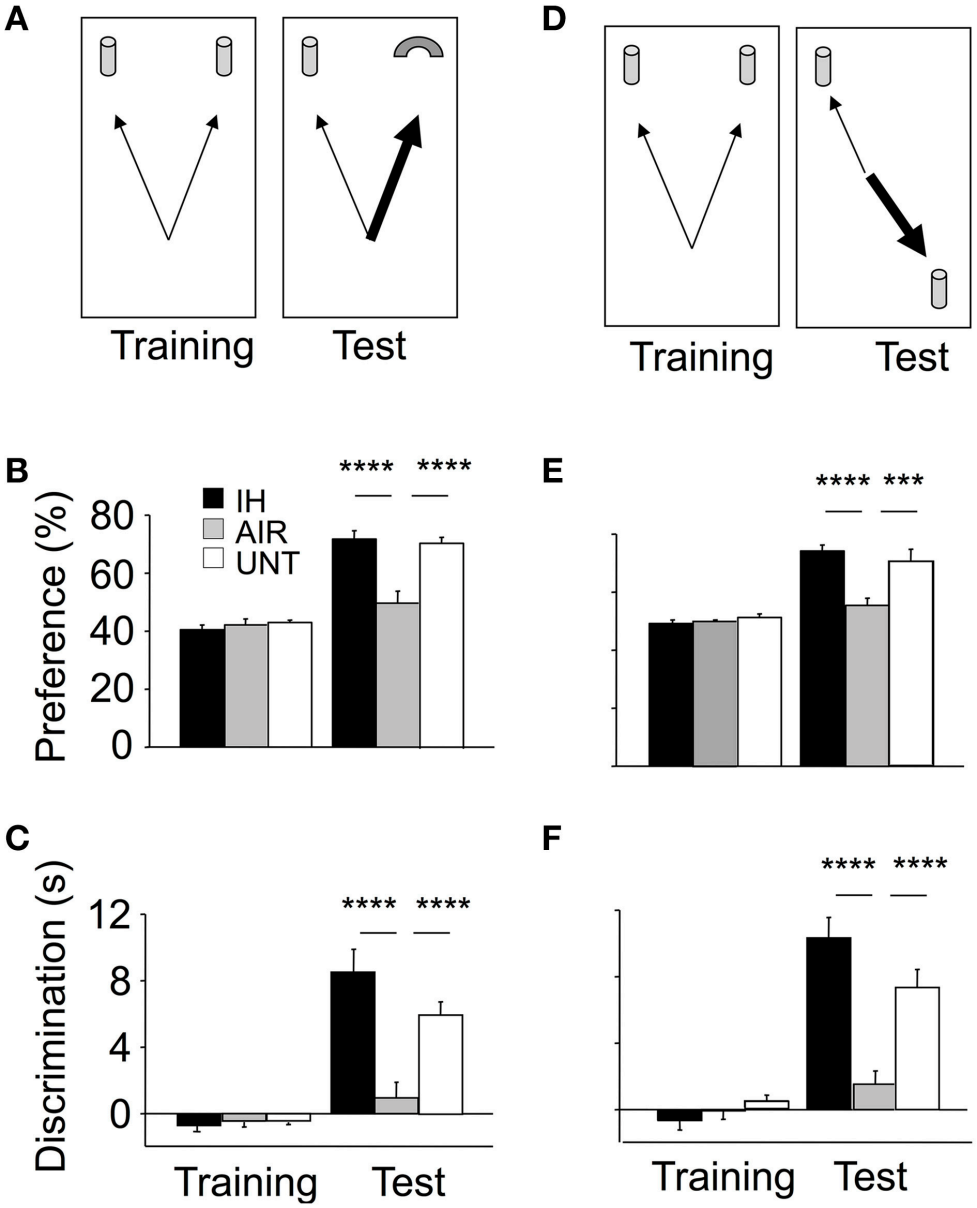
**Improvements in memory in mice postnatally exposed to IH**. Memory was assessed on P22 using the Object Recognition Task (ORT, **A–C**) and Object Location Task (OLT, **D–F**). IH, mice exposed to chronic intermittent hypoxia (*n* = 20); AIR, mice exposed to air (*n* = 20); UNT, untreated unmanipulated mice left with their dams (*n* = 19). ORT, **(B,C)** In the training phase, the three groups tended to equally explore the two objects. In the ORT test phase, the IH group had significantly higher preference **(B)** and discrimination **(C)** indices compared to the AIR group; with both values similar to those in the UNT group. OLT, **(E,F)** gave similar results. Values are group means ± SEM. ^***^p < 0.001; ^****^p < 0.0001.

Recognition memory was first evaluated using the ORT (Figure 3A). In the ORT training phase, all groups spent roughly the same time exploring the two objects (Figures 3B,C). These data suggested similar locomotion, attention, motivation, and visual perception among the groups. The ORT test phase (conducted 30 min after training) revealed significant differences across groups (main effect for group, p < 0.0001; and group-by-phase interaction, p < 0.0001; for both preference and discrimination). The IH group performed significantly better than the AIR group and similarly to the UNT group (Figures 3B,C). The OLT (Figure 3D) then confirmed these differences across groups (Figures 3E,F). During the OLT training phase, preference and discrimination indices were closely similar in the three groups. The OLT test phase revealed large and significant differences across groups: the IH group performed significantly better than the AIR group and similarly to the UNT group (main effect for group, p < 0.0001; and group-by-phase interaction, *p* < 0.0001; for both preference and discrimination; Figures 3E,F). Thus, IH prevented the adverse effects of maternal separation on memory.

### Intermittent hypoxia stimulated hippocampal neurogenesis

**Figure 4:**
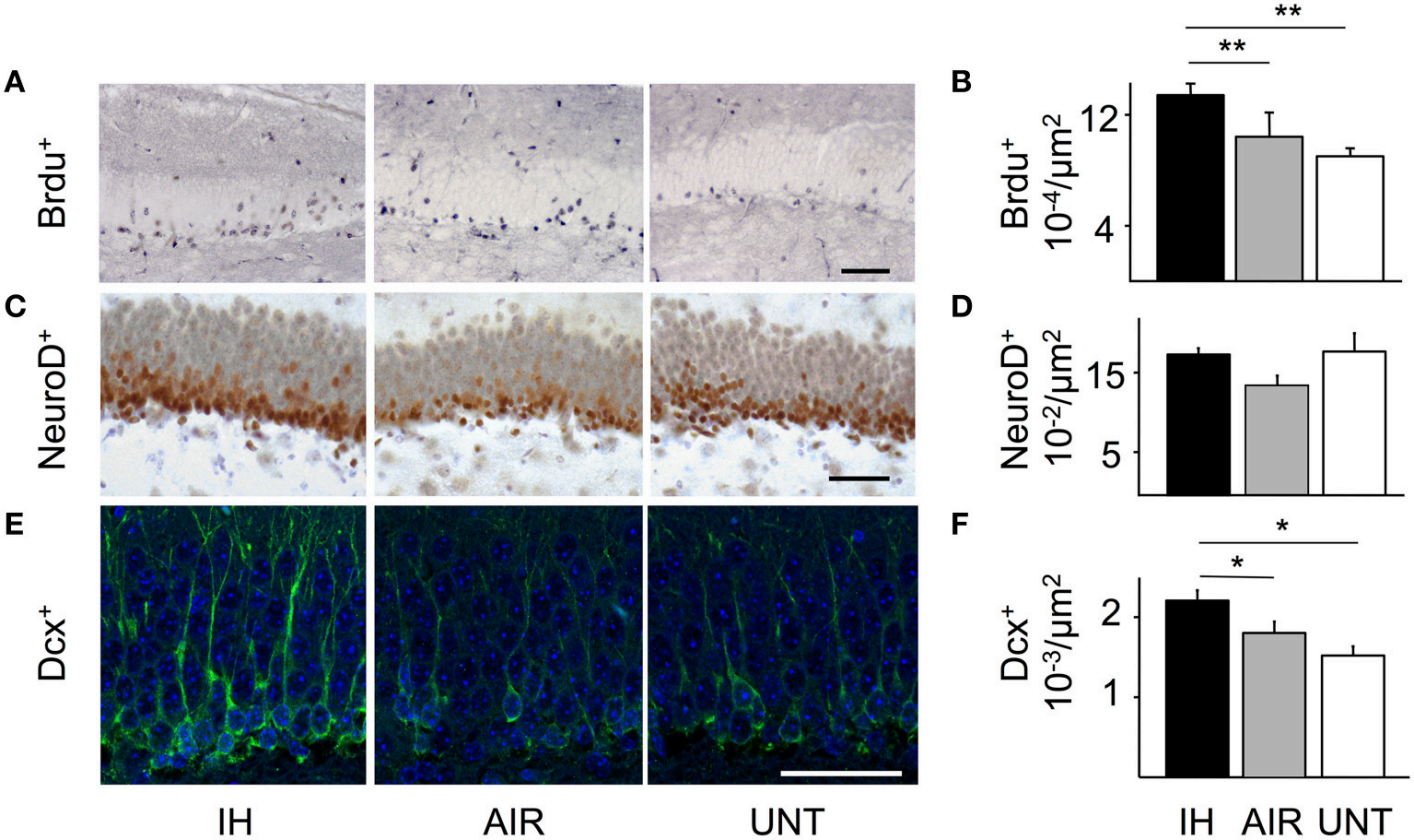
**IH-induced increase in neurogenesis**. Neurogenesis was analyzed in the dentate gyrus of mice postnatally exposed to intermittent hypoxia (IH, *n* = 22), exposed to air (AIR, *n* = 21), or left untreated with their dams (UNT, *n* = 22). **(A)** Cell proliferation was detected by BrdU labeling on P13. **(B)** Densities of BrdU-positive cells. IH increased the density of BrdU-positive cells on P13 compared to AIR. **(C)** NeuroD immunolabeling on P21. **(D)** IH tended to increase the density of NeuroD-positive cells, although the difference was not significant (IH vs. AIR: *p* = 0.055). **(E)** DCX immunolabeling on P30. **(F)** Densities of DCX-positive cells on P30. IH increased DCX-positive cell densities (IH vs. AIR group: *p* = 0.049). Scale bars = 50 μm. Values are group means ± SEM. ^*^p < 0.05, ^**^p < 0.01.

Because memory impairments are frequently associated with impaired hippocampal function, we assessed whether IH influenced neurogenesis in the dentate gyrus. The microscopic appearance and density of BrdU-positive cells revealed a proliferative effect of IH compared to AIR on P13 (main effect for group, *p* = 0.024; Figures 4A,B). Cell densities on P21 and P30 were not significantly different among groups (not shown). The proliferative effect of IH received some support from NeuroD labeling (non-significant trend: main effect for group, *p* = 0.084; Figures 4C,D) and from DCX labeling on P30 (main effect for group, *p* = 0.002; Figures 4E,F). Cell counts were not significantly different between the AIR and UNT groups at any of the study time points (Figures 4B,D,F). Other comparisons showed no significant differences (not shown). Apoptosis was hardly detectable in all three groups, as shown by their very low levels of caspase-3 activation (Supplemental Figures S1A,B). This result further supported that intermittent hypoxia increased cell proliferation, rather than decreased apoptosis.

### Intermittent hypoxia stimulated angiogenesis

**Figure 5:**
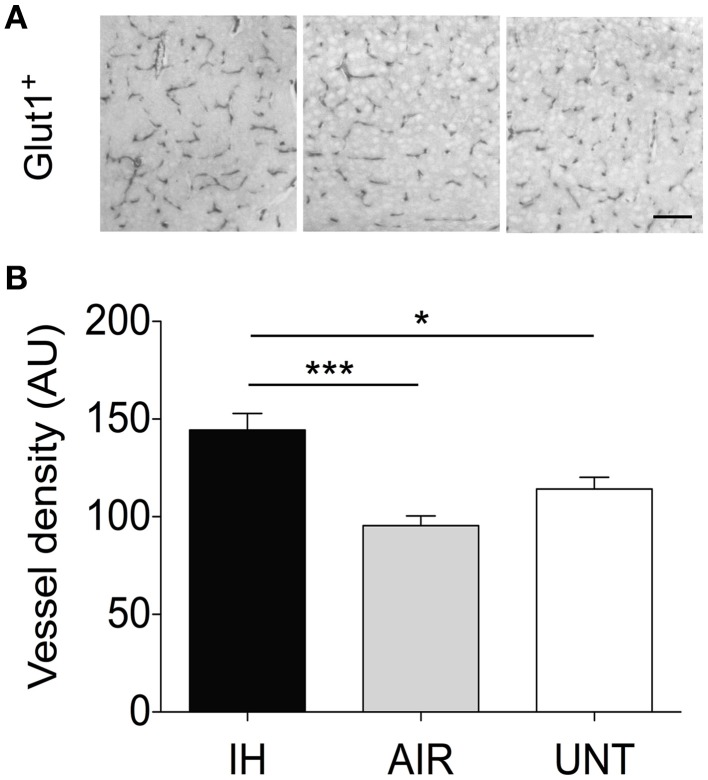
**IH-induced increase in vessel density**. Vessel density was measured in the somatosensory cortex of pups postnatally exposed to intermittent hypoxia (IH, *n* = 5), exposed to air (AIR, *n* = 9), or left untreated with their dams (UNT, *n* = 6). **(A)** Cerebral vessels in the cortex were identified by GLUT-1 immunostaining. **(B)** Vessel density analysis of GLUT-1-stained sections. IH increased angiogenesis compared to AIR and UNT. Scale bar=50 μm. Values are group means ± SEM. ^*^p < 0.05, ^***^p < 0.001.

Because angiogenesis is a common consequence of hypoxia and provides critical support for neurogenesis (as shown in mouse models of stroke, Taguchi et al., 2004), we examined whether IH exposure affected vessel density in the cerebral cortex. IH exposure significantly increased vessel density by about 40%, compared to the AIR and UNT groups (main effect for group, *p* < 0.0001, Figures 5A,B).

### Intermittent hypoxia diminished excitotoxic brain-lesion size

**Figure 6:**
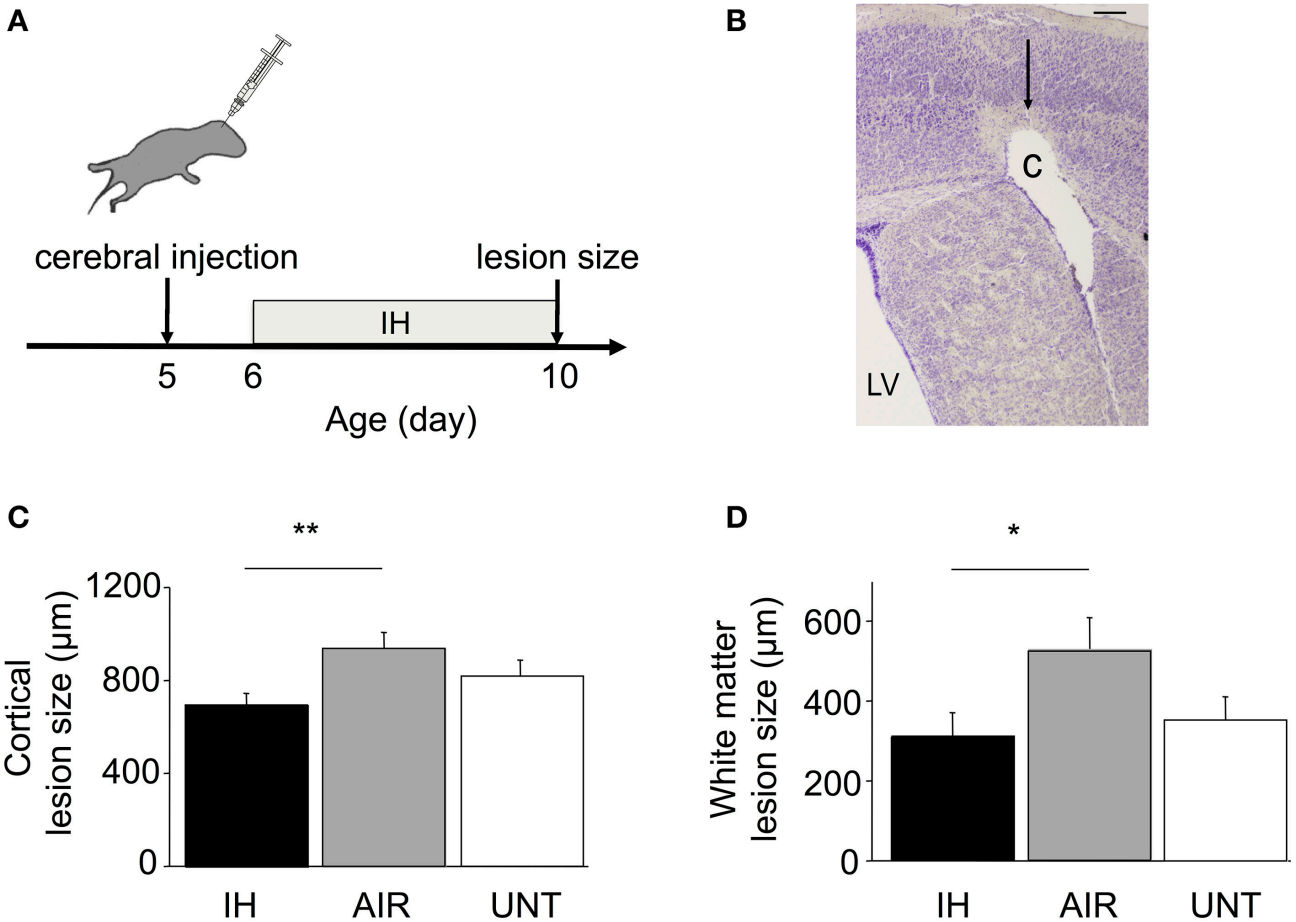
**Neuroprotection by IH in mice with excitotoxic brain lesions**. **(A)** Excitotoxic brain lesions were produced by intracerebral injections of ibotenate, a glutamate analog, on P5 in mice exposed to IH (*n* = 22), exposed to AIR (*n* = 21), or untreated and left with their dams (UNT, *n* = 22). **(B)** Coronal section of the right hemisphere showing a lesion. The brains were dissected on P10, after the end of IH exposure, and stained with cresyl violet. Ibotenate injection typically produced neuronal loss in layers III to VI (arrow) and white-matter cystic lesions **(C)**. LV, lateral ventricle. Scale bar = 40 μm. **(C,D)** Cortical and white matter lesion sizes were smaller in IH pups compared to AIR pups. Values are group means ± SEM. ^*^p < 0.05, ^**^p < 0.01.

In a separate experiment, we evaluated whether the protective effects of IH extended to excitotoxic brain lesions caused by intracerebral ibotenate injection (Figure 6A). Ibotenate injection produced excitotoxic lesions in the cortical plate and white matter in all three groups (Figure 6B). Histological analysis showed significantly smaller lesions in the IH group compared to the AIR group in the cortical plate (main effect for group, p = 0.026; Figure 6C) and, to a lesser extent, in the white matter (main effect for group, p = 0.076; not significant, Figure 6D). Brain-lesion sizes were not significantly different between the IH and UNT groups. Thus, IH provided strong protection against excitotoxic brain injury.

### Intermittent hypoxia prevented memory impairments in brain-lesioned mice

**Figure 7:**
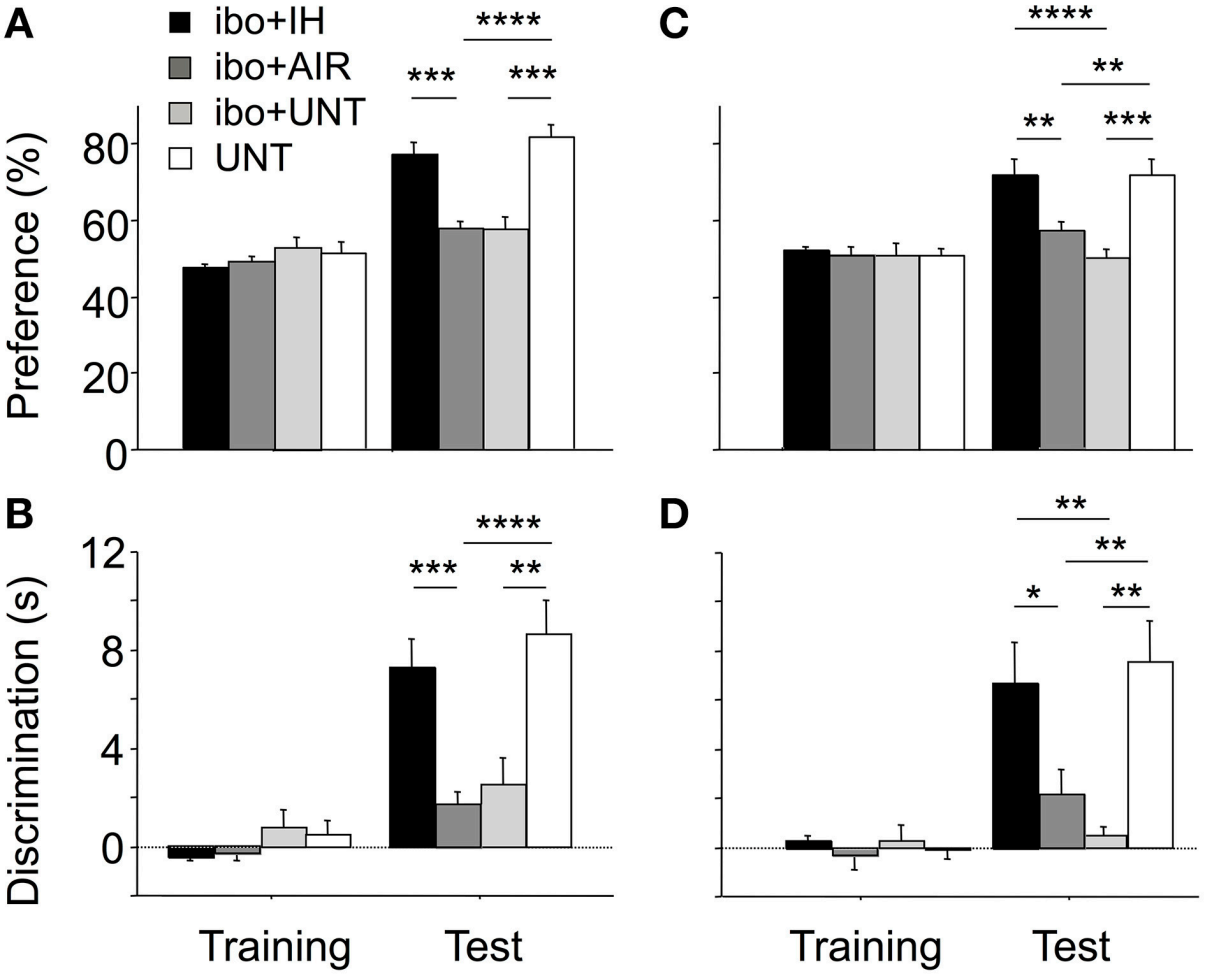
**Improvement of memory indices in brain-lesioned mice exposed postnatally to IH**. During training for ORT **(A,B)** and OLT **(C,D)**, all groups displayed similar exploration of the two objects. **(A,B)** During the ORT test phase, mice with excitotoxic brain lesions, whether unmanipulated (ibo + UNT, *n* = 15) or exposed to air (ibo + AIR, *n* = 16), displayed significantly lower indices compared to untreated pups left with their dam (UNT, *n* = 12). IH exposure prevented recognition memory dysfunction in mice with excitotoxic brain lesions (ibo + IH, *n* = 16). **(C,D)** The OLT yielded similar statistical results: IH prevented spatial memory dysfunction in mice with brain lesions. Values are group means ± SEM. ^*^p < 0.05; ^**^p < 0.01; ^***^p < 0.001; ^****^p < 0.0001.

Finally, we evaluated whether the protective effect of IH in mice with excitotoxic brain lesions extended to memory, as tested using the ORT and OLT (Figure 7). Memory indices were determined in brain-lesioned mice exposed to IH (ibo+IH), air (ibo+AIR), or no treatment (ibo+UNT) and in non-lesioned untreated mice (UNT). In the ORT training phase, all groups spent similar times exploring the two objects (Figures 7A,B). The ORT test phase revealed significant differences in memory indices among groups [main effect for group, *p* < 0.0001; and group-by-phase interaction, *p* < 0.0001; for both preference (Figure 7A) and discrimination (Figure 7B)]. Mice with brain lesions not exposed to IH showed low indices for preference (Figure 7A) and discrimination (Figure 7B) after maternal separation and air exposure (ibo+AIR) or no maternal separation (ibo+UNT). In contrast, among brain-lesioned mice, those exposed to IH (ibo+IH) showed significantly higher memory indices compared to those exposed to air (ibo+AIR) or left untreated (ibo+UNT), with values similar to those in UNT mice (Figures 7A,B). This beneficial effect of IH on memory was confirmed by the OLT (Figures 7C,D), which gave similar results to those of the ORT (preference index: main effect for group, *p* < 0.0001, and group-by-phase interaction, *p* < 0.0010; discrimination index: main effect for group, *p* < 0.0003, and group-by-phase interaction, *p* < 0.0007). Thus, IH prevented the memory impairments associated with excitotoxic brain lesions.

## Discussion

In this study, moderate IH during the early postnatal period increased cell proliferation and neurogenesis in the hippocampal dentate gyrus and improved memory. Furthermore, in mice with excitotoxic brain lesions, IH reduced lesion size and counteracted the adverse effects of brain lesions on memory. These results support the hypothesis that exposing newborn mice to moderate IH exerts neuroprotective effects.

### Intermittent hypoxia severity

Our results contrast with previous studies showing detrimental effects of IH on neurodevelopment (Gozal et al., 2001a; Row et al., 2002; Decker et al., 2003, 2005; Kheirandish et al., 2005; Douglas et al., 2007; Cai et al., 2012; Juliano et al., 2015). This difference is due to the less severe IH protocol used in our study compared to previous studies. We exposed the pups to IH from P6 to P10, i.e., before the period of vulnerability to IH (P10–P25; Gozal et al., 2001a). Furthermore, IH exposure was much shorter (5 days) than in most previous studies: 2 weeks starting on P1 (Juliano et al., 2015) or P10 (Kheirandish et al., 2005), 3 weeks starting on P10 (Row et al., 2002), or 4 weeks starting on P2 (Douglas et al., 2007), with F_I_O_2_ values (10% O_2_) similar to those in our study. In the few studies that used IH-exposure periods (P7–P11) and F_I_O_2_ values (6–8% O_2_) similar to those in our study (Decker et al., 2003, 2005), the frequency of hypoxic events was considerably higher (60 vs. 20 events/h). Furthermore, IH exposure duration was 6 h/day in our study compared to 12 h (Kheirandish et al., 2005) or 24 h (Gozal et al., 2001b; Row et al., 2002; Douglas et al., 2007) in earlier studies. Thus, although circumspection is in order when making comparisons based on multiple criteria, our IH protocol seems substantially milder than the protocols used in studies that demonstrated detrimental effects of IH.

Our IH protocol induced some of the main features of moderately severe AOP. The repetitive oxyhaemoglobin desaturation events occurred during a period corresponding to the occurrence of AOP in humans. The magnitude of desaturation was within the range of clinically significant values in preterm infants (e.g., 80% for ≥10 s, Di Fiore et al., 2012). The frequency of hypoxic events (< 85% SaO_2_, 120/day) was similar to values reported in preterm infants at 24–27 weeks gestational age (Di Fiore et al., 2012). IH was associated with moderate changes in HR. This result is consistent with a study in which 2-day-old and 8-day-old pups exposed to IH (3% F_I_O_2_ for about 2 min) showed no significant changes in HR, whereas bradycardia was observed from P10 onwards (Chintamaneni et al., 2013). Similarly, in a recent study in P5 mouse pups, an IH protocol that decreased SpO_2_ to 60% was not associated with bradycardia (Juliano et al., 2015). Our results indicate clearly that HR increased in response to hypoxia. Thus, our model replicated the desaturation spells associated with apneas, without the attendant bradycardia (a frequent event in infants; Ramanathan et al., 2001).

### Intermittent hypoxia improved memory

The ORT and OLT have been increasingly used in recent years due to their sensitivity and simplicity (Akkerman et al., 2012). Both tests assess recognition memory, although they differ regarding the importance of the spatial component (which is prominent in the OLT) and, therefore, provide complementary information on memory abilities. Maternal separation impaired memory in mice, as previously reported in rat pups (Hulshof et al., 2011). Our main and novel finding is that the adverse effects of maternal separation on memory were counteracted by IH, thus leading to similar indices in IH and UNT pups. Our findings constitute the first evidence that an IH protocol mimicking AOP may improve important dimensions of cognitive performance.

### Intermittent hypoxia induced neurogenesis

In rodents, the hippocampus is critical for the object-place associations in OLT (Barker and Warburton, 2011), whereas the prefrontal cortex and rhinal cortex are closely involved in responses to the ORT (Li et al., 2011). We found that IH stimulated neurogenesis in the hippocampus, compared to air exposure. As a rule, conditions that increase memory performance also stimulate hippocampal neurogenesis, and conditions that reduce neurogenesis are associated with cognitive impairments (Pourié et al., 2006), suggesting a causal link between increased neurogenesis and improved memory in our study.

### Intermittent hypoxia induced neuroprotection against brain lesions

The neuroprotective effects of IH were confirmed in the experiment combining maternal separation with brain lesions resembling those observed in preterm infants (Ramanantsoa et al., 2013). IH exposure after ibotenate injection significantly reduced both cortical and white matter lesion sizes, compared to air exposure. This result extended previous results showing neuroprotective effects of hypoxia (Mateika et al., 2015). For example, exposing 4-day-old mice to continuous hypoxia (8% O_2_) for 3 h before ibotenate injection decreased brain-lesion size by about 30% (Laudenbach et al., 2007). Bouts of continuous moderate postnatal hypoxia each lasting several hours improved long-term hippocampal potentiation and memory (Zhang et al., 2005; Lu et al., 2009). Furthermore, a single 5-min episode of anoxia on the day of birth stimulated brain neurogenesis and improved memory (Pourié et al., 2006; Martin et al., 2010). However, the IH patterns used in these studies were characterized by single sessions per day or by exposures ranging from minutes to several hours and therefore departed considerably from the apnea-like IH pattern. Our results constitute the first evidence that an IH protocol mimicking AOP may afford neuroprotection. The concept that “low dose” IH may be a simple, safe, and effective treatment with considerable therapeutic potential for multiple clinical disorders is receiving increasing support from experimental and clinical studies (Almendros et al., 2014; Navarrete-Opazo and Mitchell, 2014; Mateika et al., 2015). The present results extend this concept to AOP.

### Clinical implications

One limitation of the present study is that IH was delivered to healthy mouse pups, whereas AOP in preterm human infants is frequently accompanied by other common perinatal complications of prematurity. One such common complication is inflammation, which contributes strongly to AOP (Di Fiore et al., 2013) and also contributes to brain damage and neurodevelopmental disturbances (Hagberg et al., 2015). AOP itself can sustain a proinflammatory cascade (Martin et al., 2011). Therefore, a crucial issue is determining whether inflammation could undermine IH-induced neuroprotection. In support of this possibility, previous studies in adult rats have shown that even moderate inflammation induced by lipopolysaccharide injections disrupts IH-induced ventilatory long-term facilitation, another form of neuronal plasticity resulting from IH (Huxtable et al., 2013). Furthermore, in rats with spinal cord injuries, inflammation counteracts the functional recovery induced by IH treatment (Huxtable et al., 2015). Moreover, IH treatments that improve functional recovery in rats with spinal cord injuries (Gonzalez-Rothi et al., 2015) do not increase microglial inflammatory gene expression in any central nervous system region studied (Peters et al., 2015). These previous studies strongly suggest the need for experiments to determine whether the absence of inflammation in the central nervous system is a necessary condition for the neuroprotective effects of IH in newborn rodents. Also, the present study did not address the effects of IH in situations where supplemental oxygen is delivered. Treatment-induced hyperoxia combined with AOP-induced IH is common in preterm infants (Coste et al., 2015). The resulting fluctuation between high and low oxygenation levels may promote retinopathy of prematurity (Penn et al., 1994).

Despite their limitations, our results are relevant to the debate about whether AOP requires treatment. For decades, neonatal caffeine has been an important part of the routine management of AOP. A randomized trial in preterm infants showed that caffeine reduced the risks of bronchopulmonary dysplasia, severe retinopathy of prematurity, cerebral palsy, and cognitive delay at 18 months of age; and improved gross motor function at 5 years of age (Schmidt et al., 2012). The neuroprotective effect of caffeine at 18 months was largely explained by the earlier discontinuation of positive airway pressure therapy. However, the influence of AOP severity on these effects was not evaluated in this trial. Our study emphasizes the need for stratifying data by AOP severity in clinical trials of caffeine or other methylxanthines. If IH exerts neuroprotective effects in a subgroup of patients with moderate apneas, as suggested by our study, caffeine may be of little or no benefit in those patients, unless it conveys neuroprotection via different mechanisms such as anti-inflammatory effects (Köroğlu et al., 2014).

## Funding

This study was supported by grants from the ANR (the French national research agency, Oxyprem) and DHU PROTECT. Myriam Bouslama received a doctoral grant from the Paris-Est Créteil University, a grant from the UNESCO-L'ORÉAL Fellowship for Women in Life Sciences, and a grant from the Association for the Study of Pediatric Pathology.

### Conflict of interest statement

The authors declare that the research was conducted in the absence of any commercial or financial relationships that could be construed as a potential conflict of interest.
