# Corrigendum: Protective effects of intermittent hypoxia on brain and memory in a mouse model of apnea of prematurity

**DOI:** 10.3389/fphys.2016.00105

**Published:** 2016-03-22

**Authors:** Myriam Bouslama, Homa Adle-Biassette, Nelina Ramanantsoa, Thomas Bourgeois, Bieke Bollen, Olivier Brissaud, Boris Matrot, Pierre Gressens, Jorge Gallego

**Affiliations:** ^1^Inserm, U1141, Robert Debré HospitalParis, France; ^2^Paris Diderot-Paris 7 UniversityParis, France; ^3^Department of Pathology, Lariboisière Hospital, AP-HPParis, France; ^4^Laboratory of Biological Psychology, University of LeuvenLeuven, Belgium; ^5^Neonatal Intensive Care Unit, Bordeaux University HospitalBordeaux, France

**Keywords:** sleep disordered breathing, neurogenesis, brain injury, cognitive dysfunction, control of breathing

Due to an error in the proofs missed by the authors, the name of the author Homa Adle-Biassette was reported incorrectly as Homa Adla-Biassette. Also the citation was Adla-Biassette H instead of Adle-Biassette H.

The affiliation for Dr. Adle-Biassette was incomplete and should be Department of Pathology, Lariboisière Hospital, AP-HP, Paris, France.

The authors apologize for these mistakes. The errors do not affect the scientific results of the article in any way.

The original article has been updated.

## Author contributions

All authors listed, have made substantial, direct and intellectual contribution to the work, and approved it for publication.

### Conflict of interest statement

The authors declare that the research was conducted in the absence of any commercial or financial relationships that could be construed as a potential conflict of interest.

