# Supplementary material for: Protective effects of intermittent hypoxia on brain and memory in a mouse model of apnea of prematurity
Source: Front Physiol. 2015 Nov 4;6:313. doi: 10.3389/fphys.2015.00313 (PMC4631942; doi:10.3389/fphys.2015.00313)
Supplement: Supplementary file 1 [file Image1.PDF]

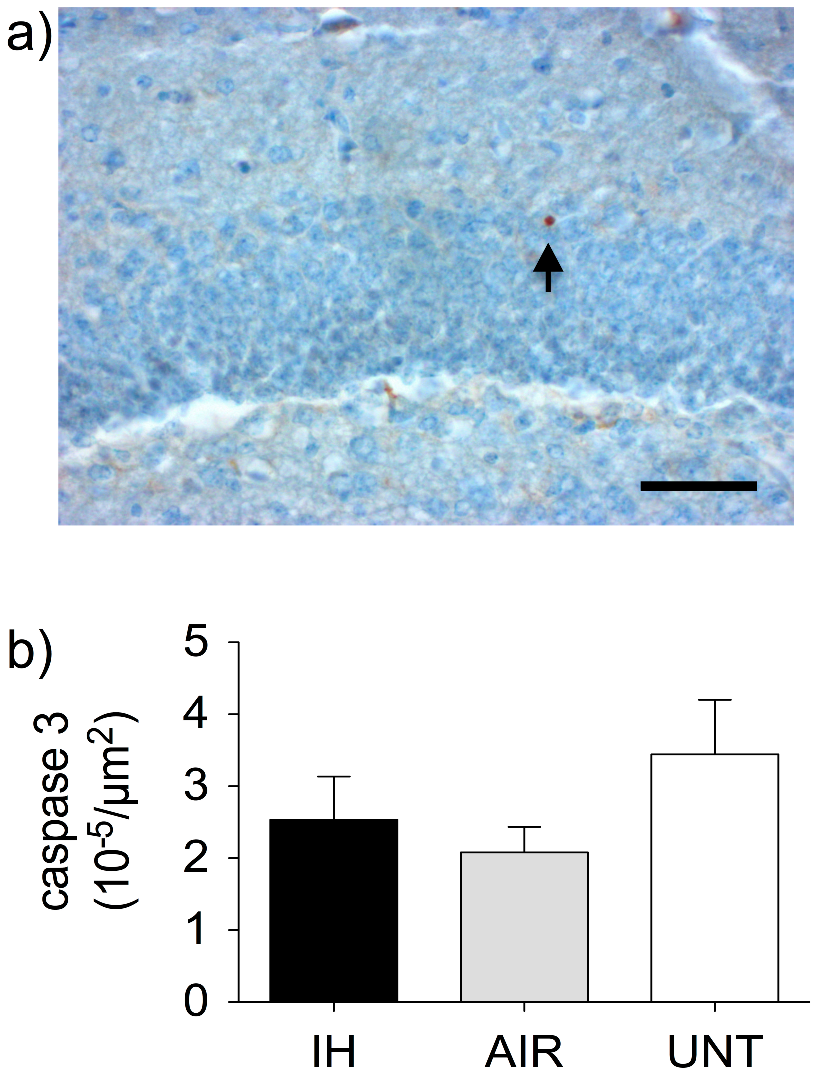

Supplemental Figure S1: Apoptosis. Caspase-3 immunolabeling was analyzed in the dentate gyrus of mice postnatally exposed to intermittent hypoxia (IH, n=6), exposed to air (AIR, n=8), or left untreated with their dams (UNT, n=8) by caspase-3 labeling on P13. a) Caspase-3 positive cells (arrow) were hardly detected in all three groups. b) Caspase-3 positive cell densities were not significantly different in the three groups. Scale bars =50 mm.
